# Occurrence of tick-transmitted pathogens in dogs in Jos, Plateau State, Nigeria

**DOI:** 10.1186/1756-3305-7-119

**Published:** 2014-03-24

**Authors:** Mathew Adamu, Milana Troskie, David O Oshadu, Dikeledi P Malatji, Barend L Penzhorn, Paul T Matjila

**Affiliations:** 1 Department of Veterinary Parasitology and Entomology, College of Veterinary Medicine, University of Agriculture Makurdi, P.M.B. 2373, Makurdi, Nigeria; 2 Department of Veterinary Tropical Diseases, Faculty of Veterinary Science, University of Pretoria, Pretoria, South Africa; 3 Department of Wildlife and Animal Science, Faculty of Natural and Agricultural Science, University of Pretoria, Pretoria, South Africa; 4 Department of Life and Consumer Sciences, College of Agriculture and Environmental Sciences, Unisa, Florida, South Africa

**Keywords:** *Babesia rossi*, *BrEMA1*, *Haemaphysalis leachi*, *Haemaphysalis elliptica*, *Rhipicephalus sanguineus*, Domestic dogs, Nigeria

## Abstract

**Background:**

Canine babesiosis caused by *Babesia rossi*, transmitted by *Haemaphysalis elliptica* in South Africa, has also been reported from Nigeria. Although *H. leachi* (sensu lato) is widespread in sub-Saharan Africa, published literature on the occurrence of canine babesiosis is meagre. It has been postulated that the genotype of *Babesia rossi* Erythrocyte Membrane Antigen 1 (*BrEMA*1) may be linked to virulence of the specific isolate. The primary objective of this study was to detect and characterise tick-borne pathogens in dogs presented to a veterinary hospital using molecular techniques. In *B. rossi*-positive specimens, we aimed to determine whether the *BrEMA*1 gene occurred and to compare genotypes with those found in other isolates. Lastly, we wished to identify the tick species that were recovered from the sampled dogs.

**Methods:**

Blood specimens (n = 100) were collected during January to March 2010 from domestic dogs presented at an animal hospital in Jos, Plateau State, Nigeria. They were screened for the presence of *Babesia*/*Theileria* and *Ehrlichia*/*Anaplasma* genomic DNA using PCR and Reverse Line Blot (RLB) assays. Positive *B. rossi* specimens were tested for the presence of the *BrEMA1*gene using an RT-PCR. In addition, ticks were collected from dogs found to be infested during sampling.

**Results:**

On RLB, 72 (72%) of the specimens were positive for one or more haemoparasites. Of the positive specimens, 38 (53%) were infected with *B. rossi;* 9 (13%) with *Theileria* sp. (sable); 5 (7%) with either *Ehrlichia canis* or *Anaplasma* sp. Omatjenne, respectively; 3 (4%) with *Theileria equi*; and 1 (1%) with *B. vogeli* and *E. ruminantium*, respectively. Co-infections were detected in 13 (18%) of the specimens. Results of RT-PCR screening for the *BrEMA1* gene were negative. A total of 146 ticks belonging to 8 species were collected and identified: *Rhipicephalus sanguineus* 107 (73%), *Haemaphysalis leachi* (sensu stricto) 27 (18%), *R. turanicus* 3 (2%), and *Amblyomma variegatum*, *H. elliptica, R. lunulatus, R. muhsamae* and *R. senegalensis* 1 (1%), respectively.

**Conclusions:**

Up to 8 tick-borne pathogens possibly occur in the dog population at Jos, with *B. rossi* being the most prevalent. The absence of the *BrEMA1* gene suggests that *B. rossi* occurring in that area may be less virulent than South African isolates.

## Background

Tick-borne pathogens remain an important cause of disease among canine populations world-wide. Canine babesiosis caused by *Babesia rossi* is the most common and economically important tick-borne disease in South Africa [1], where the known vector is *Haemaphysalis elliptica* (formerly lumped with *H. leachi*) [2]. Presence in Africa of the less virulent *Babesia vogeli*, transmitted by *Rhipicephalus sanguineus*, was confirmed in 2004 [3]. Although *H. leachi* (sensu lato) is a ubiquitous tick of tropical and southern Africa [2,4], the published literature on the occurrence of canine babesiosis in Africa is surprisingly meagre. Apart from South Africa, where the disease has been studied intensively, and Nigeria, the only published references traced were from Zimbabwe [5], Zambia [6,7], the Sudan [8] and the Cape Verde islands [9].

In Nigeria, canine babesiosis was first mentioned in an annual report of the Veterinary Department in 1926 [10]. The disease occurred more frequently in imported dogs, while puppies born in the country, especially those of indigenous breeds, developed the disease in a milder form and usually recovered. This situation has persisted over many years [11]. In a survey of 400 dogs sampled randomly from many parts of Nigeria, only eight dogs (2.3%) were positive for *B. rossi*, while a single dog was positive for *B. vogeli*[12]. Blood smears made from 500 dogs presented to veterinary clinics in Ibadan, Oyo State, were examined microscopically; 53 (26.0%) were found to be infected with *B. canis* (sensu lato), while 41 (20.2%) were infected with *B. gibsoni*[13]. *Babesia canis* (sensu lato) was reported from Zaria, Kaduna State [14,15]. A low prevalence (2.8%) of *B. canis* (sensu lato) infection was found in a blood-smear-based survey among slaughtered dogs in Maiduguri, Borno State [16]. Using molecular detection and characterisation on blood specimens of 181 dogs presented to veterinary hospitals in four states, *B. rossi* was detected in 2/17 dogs (11.8%) in Rivers State, while in Plateau State it was found in 6/41 (14.6%) dogs in Jos North and in 4/64 (4.8%) dogs in Jos South [17]. A single dog in Kaduna state was found to be positive for *B. vogeli*[17]. Interestingly, *B. canis* (sensu stricto) and *B. rossi* co-infection was found in a dog that had never left Vom, Plateau State [18]. This stimulated renewed interest in the epidemiology of canine babesiosis in Africa, as it was the first confirmation of the occurrence of *B. canis* in a geographical region were *Dermacentor reticulatus*, the only confirmed vector of *B. canis*, does not occur [18].

Clinical signs of canine babesiosis always include fever and splenomegaly, while inappetence, weakness, lethargy, generalised lymphadenopathy, anaemia and haemoglobinuria due to erythrolysis may also occur [19]. In some cases infection remains sub-clinical [20]. The clinical manifestation of *B. rossi* infection is classified as either uncomplicated or complicated [21,22]. It is regarded as uncomplicated if the clinical signs can be attributed solely to mild or moderate anaemia [21]. On the other hand, complicated cases are those where there is evidence of non-solid-organ failure characterised by severe anaemia and haemoconcentration or organ dysfunction/failure [22]. The mechanisms resulting in *B. rossi* being associated with such diverse clinical manifestations remain unknown. One possibility is that it may be due to genotypic differences among *B. rossi* strains, as has been suggested for *B. canis* (sensu stricto) [23]. A polymorphic phosphoprotein localised on the cytoplasmic surface of *B. rossi*-infected erythrocytes has been named *Babesia rossi* erythrocyte membrane antigen 1 (*BrEMA1*) [19]. *BrEMA1* genes of various laboratory strains code for polymorphic proteins that contain various numbers of repetitive hexapeptide motifs [19]. The exact function of this gene is unknown, but it is hypothesised that it may be related to virulence [19].

The primary objective of this study was to detect and characterise tick-borne pathogens in dogs presented to a veterinary hospital in Jos, Plateau State, Nigeria, using molecular techniques (Polymerase Chain Reaction and Reverse Line Blot). In *B. rossi*-positive specimens,we aimed to determine whether the *BrEMA*1 gene occurred and to compare genotypes with those found in other isolates. Lastly, we wished to identify the tick species that were present on the sampled dogs.

## Methods

### Collection of samples

The study was carried out on dogs presented for treatment at the Evangelical Church Winning All (ECWA) Animal Hospital at Jos, Plateau State, Nigeria (9°56’N, 8°53’E; altitude 1217 m), virtually in the geographical centre of Nigeria. A total of 100 dogs were examined over a 3-month period (Jan-Mar 2010): 55 females and 43 males; in 2 cases the gender/age were not recorded. Of the 55 female dogs, 41 were < 18 months old and 14 were regarded as adult. Of the 43 male dogs, 25 were < 18 months old and 18 were regarded as adults. Dogs were clinically examined upon presentation. If detected, ticks were collected, stored in 70% alcohol and sent to the Department of Veterinary Tropical Diseases, Faculty of Veterinary Science, University of Pretoria, South Africa, for identification by stereomicroscopy [24]. Blood specimens were collected into EDTA tubes from the cephalic vein. From the EDTA tubes 80 μl of blood were micro-pipetted onto the Whatman FTA classic card (GE Healthcare UK Limited). These cards were dried and sent to the Department of Veterinary Tropical Diseases of the Faculty of Veterinary Science University of Pretoria, South Africa.

### DNA extraction

DNA was extracted from the filter paper specimen. The QI Amp blood and tissue kit (Qiagen, Hilden, Germany) was used for DNA extractions following the manufacturer’s protocols.

### PCR

The *Babesia/Theileria* PCR was performed with primers RLB-F2 (5’-GAC ACAGGG AGG TAG TGA CAA G-3’) and RLB-R2 (biotin-5’-CTA AGA ATT TCA CCT CTG ACA GT-3’) amplifying a fragment of 460–540 bp from the 18S rRNA gene spanning the V4 region [3,25]. The *Ehrlichia/Anaplasma*PCR was performed with primers Ehr-R (5’-biotin-CGG GAT CCC GAG TTT GCC GGG ACT TYT TCT-3’) and Ehr-F (50-GGA ATT CAG AGT TGG ATC MTG GYT CAG-30) amplifying a fragment of 460–520 bp from the V1 hypervariable region of the 16S SSU rRNA gene [26,27]. The conditions for the PCR included an initial step of 3 min at 42°C, 10 min at 94°C, 10 cycles of 94°C (20 s)–67°C (30 s)–72°C (30 s), with lowering of the annealing step after every second cycle by 2°C (touchdown PCR). The reaction was then followed by 40 cycles of denaturation at 94°C for 30 s, annealing at 57°C for 30 s and extension at 72°C for 30 s.

### Reverse line blot hybridisation

RLB was subsequently conducted on amplified products (*Babesia, Theileria, Anaplasma* and *Ehrlichia*) as previously described [3]. The probes and their sequences used for detecting pathogen DNA are listed in Table 1. Each probe on the membrane has a related cloned positive control that is included with every RLB test.

**Table 1:** List of organisms and their corresponding probe sequences used to detect pathogen DNA

*Anaplasma bovis*	GTA GCT TGC TAT GRG AAC A
*Anaplasma centrale*	TCG AAC GGA CCA TAC GC
*Anaplasma marginale*	GAC CGT ATA CGC AGC TTG
*Anaplasma ovis*	ACC GTA CGC GCA GCT TG
*Anaplasma phagocytophilum* 1	TTG CTA TAA AGA ATA ATT AGT GG
*Anaplasma* sp. (Omatjenne)	CGG ATT TTT ATC ATA GCT TGC
*Babesia bicornis*	TTG GTA AAT CGC CTT GGT C
*Babesia bigemina*	CGT TTT TTC CCT TTT GTT GG
*Babesia bovis*	CAG GTT TCG CCT GTA TAA TTG AG
*Babesia caballi*	GTG TTT ATC GCA GAC TTT TGT
*Babesia canis*	TGC GTT GAC CGT TTG AC
*Babesia* catch-all 1	ATT AGA GTG TTT CAA GCA GAC
*Babesia* catch-all 2	ACT AGA GTG TTT CAA ACA GGC
*Babesia divergens*	ACT RAT GTC GAG ATT GCA C
*Babesia felis*	TTA TGC GTT TTC CGA CTG GC
*Babesia gibsoni*	CAT CCC TCT GGT TAA TTT G
*Babesia leo*	ATC TTG TTG CCT TGC AGC T
*Babesia microti*	GRC TTG GCA TCW TCT GGA
*Babesia occultans*	CCT CTT TTG GCC CAT CTC GTC
*Babesia ovis*	TGC GCG CGG CCT TTG CGT T
*Babesia rossi*	CGG TTT GTT GCC TTT GTG
*Babesia* sp. (sable)	GCT GCA TTG CCT TTT CTC C
*Babesia vogeli*	AGC GTG TTC GAG TTT GCC
*Ehrlichia/Anaplasma* catch-all	GGG GGA AAG ATT TAT CGC TA
*Ehrlichia canis/Ehrlichia ovina*	TCT GGC TAT AGG AAA TTG TTA
*Ehrlichia chaffeensis*	ACC TTT TGG TTA TAA ATA ATT GTT
*Ehrlichia ruminantium*	AGT ATC TGT TAG TGG CAG
*Theileria annae*	CCG AAC GTA ATT TTA TTG ATT TG
*Theileria annulata*	CCT CTG GGG TCT GTG CA
*Theileria/Babesia* catch-all	TAA TGG TTA ATA GGA RCR GTT G
*Theileria bicornis*	GCG TTG TGG CTT TTT TCT G
*Theileria buffeli*	GGC TTA TTT CGG WTT GAT TTT
*Theileria* catch-all	ATT AGA GTG CTC AAA GCA GGC
*Theileria equi*	TTC GTT GAC TGC GYT TGG
*Theileria lestoquardi*	CTT GTG TCC CTC CGG G
*Theileria mutans*	CTT GCG TCT CCG AAT GTT
*Theileria ovis*	TTG CTT TTG CTC CTT TAC GAG
*Theileria parva*	GGA CGG AGT TCG CTT TG
*Theileria separata*	GGT CGT GGT TTT CCT CGT
*Theileria* sp. (buffalo)	CAG ACG GAG TTT ACT TTG T
*Theileria* sp. (duiker)	CAT TTT GGT TAT TGC ATT GTG G
*Theileria* sp. (kudu)	CTG CAT TGT TTC TTT CCT TTG
*Theileria* sp. (sable)	GCT GCA TTG CCT TTT CTC C
*Theileria taurotragi*	TCT TGG CAC GTG GCT TTT
*Theileria velifera*	CCT ATT CTC CTT TAC GAG T

### Real-time PCR

Primers Frep*BrEMA*1 (5’-CCA ACA TTG ATG ATG ACA A-3’) and Rrep*BrEMA*1 (5’-CTG CAT GTC AGC TTC ATC A-3’) for real-time PCR were used to specifically amplify the Br*EMA*1 gene on samples that tested positive for *B. rossi* on RLB. The eBox Light Cycler DNA Master SYBR Green 1 Kit was used (Roche Diagnostics). The real-time PCR reaction mixture consisted of 13.4 μl of water PCR-grade, 1.6 μl MgCl_2_ (5 pmol), 1 μl (5 pmol) of each primer, 2 μl Light Cycler® DNA Master SYBR Green I and 1 μl of DNA to a total volume of 19 μl. The real-time PCR was performed in 32 capillaries. Light Cycler 2.0 (Roche Diagnostics) was used to amplify genomic DNA.

### Sequencing

Two samples (RLB 42 and 67) that tested positive on RLB for *B. rossi* were randomly selected for 18S rRNA gene sequencing. The full-length 18S rRNA gene of both samples was amplified using 20 pmol of primers Nbab-1 F (5’-AAGCCATGCATGTCTAAGTATAAGCTTTT-3’) and reverse primer TB 18S-Rev (5’-GAATAATTCACCGGATCACTCG-3’) [28,29] to give a PCR amplicon of *ca*. 1700 base pairs. PCR amplification was done with 2.5 μl of extracted DNA in a final volume of 25 μl of PCR reaction containing 10 μl PCR grade water, 10 μM of each primer, and 12.5 μl of High Fidelity PCR Master. The cycling conditions consisted of an initial cycle of 5 min at 94°C, 30 cycles of amplification (94°C for 30 sec, 55°C for 30 sec and 72°C for 1 min) and 1 cycle of 7 min at 72°C. The PCR products were purified using the QIAquick PCR Purification Kit (Qiagen, Germany) and sent to a commercial laboratory (Inqaba Biotech) for sequencing.

### Phylogenetic analysis

Sequence data for the full-length 18S rRNA gene were assembled and edited to a total length of 1532 bp using GAP4 of the Staden package (Version 1.6.0 for Windows) and deposited in Genbank [30]. The sequences were aligned with sequences of related genera using ClustalX (Version 1.81 for Windows). The alignments were adjusted manually using the BioEdit program (version 7.0.5.2) [31]. Two generated sequences and 18 Genbank sequences with 1532 characters where analysed. A phylogenetic tree was generated using the ‘GeneBee’ ( http://www.//genebee.msu.su/genebee.html) programme. Phylogenetic analyses using cluster distance algorithm method were carried out (Phylip, Multiline) into the program format from the aligned nucleotide sequences. In the cluster algorithm, the notion of distance between groups of sequences was used for setting the branching order ( http://www.//genebee.msu.su/genebee.html).

### Nucleotide sequence accession numbers

The 18S rRNA gene sequences obtained from this study were submitted to GenBank under the following accession numbers: *B. rossi* RLB42: JQ 613104 and *B. rossi* RLB67: JQ 613105, respectively.

## Results

The RLB results showed that 72 (72%) of the specimens were positive for one or more haemoparasites (Table 2). In the 72 infected dogs, single infections occurred in 49 (68.1%) and co-infections occurred in 23 (31.9%). Seven tick-transmitted pathogens were detected, while further undescribed species or species variants were also captured: in 6/72 (8%) specimens by the *Theileria/Babesia* catch-all probe and in 4/72 (6%) specimens by the *Ehrlichia*/*Anaplasma* catch-all probe. *Babesia rossi*, present in 53% of the dogs, was by far the more prevalent pathogen. Non-specific clinical signs such as inappetance, pyrexia, pale mucous membranes, rough hair coat, emaciation and weakness were observed, with 90% of the dogs showing 2 or 3 of these clinical signs. None of the dogs exhibited clinical signs associated with severe or complicated canine babesiosis.

**Table 2:** Number of dogs infected with single or multiple tick-borne pathogens

**Nature of infection**										
	***B. rossi***	***B. vogeli***	***E. canis***	***E. ruminantium***	***A*. sp. (Omatjenne)**	***T. equi***	***T. *** **sp. (sable)**	**B/T**	**E/A**	**Total**
Single infection	31	0	1	0	4	1	8	3	1	49
Co-infection with:										
*B. rossi*	-	1	3	0	0	2	1	-	1	8
*B. vogeli*	1	-	0	0	0	0	0	-	0	1
*E. canis*	3	0	-	1	0	0	0	0	-	4
*E. ruminantium*	0	0	1	-	0	0	0	0	-	1
*A*. sp. (Omatjenne)	0	0	0	0	-	0	0	1	-	1
*T. equi*	2	0	0	0	0	-	0	-	0	2
*T*. sp. (sable)	1	0	0	0	0	0	-	-	2	3
B/T	-	-	0	0	1	-	-	-	0	1
E/A	0	0	-	-	-	0	0	2	-	2
**Total**	**38**	**1**	**5**	**1**	**5**	**3**	**9**	**6**	**4**	**72**

*B. rossi = Babesia rossi*; *B. vogeli = Babesia vogeli*; *E. canis = Ehrlichia canis*; *E. ruminantium = Ehrlichia ruminantium*; *A*. sp. (Omatjenne) = *Anaplasma* species (Omatjenne); *T. equi = Theileria equi*; *T*. sp. (sable) = *Theileria* species (sable); B/T = *Babesia/Theileria* catch-all; E/A = *Ehrlichia/Anaplasma* catch-all.

### *BrEMA1*Real Time- PCR

Real-time PCR did not detect the *BrEMA*1 gene in samples that were *Babesia rossi-*positive on RLB.

### Sequencing and phylogenetic analysis

**Figure 1:**
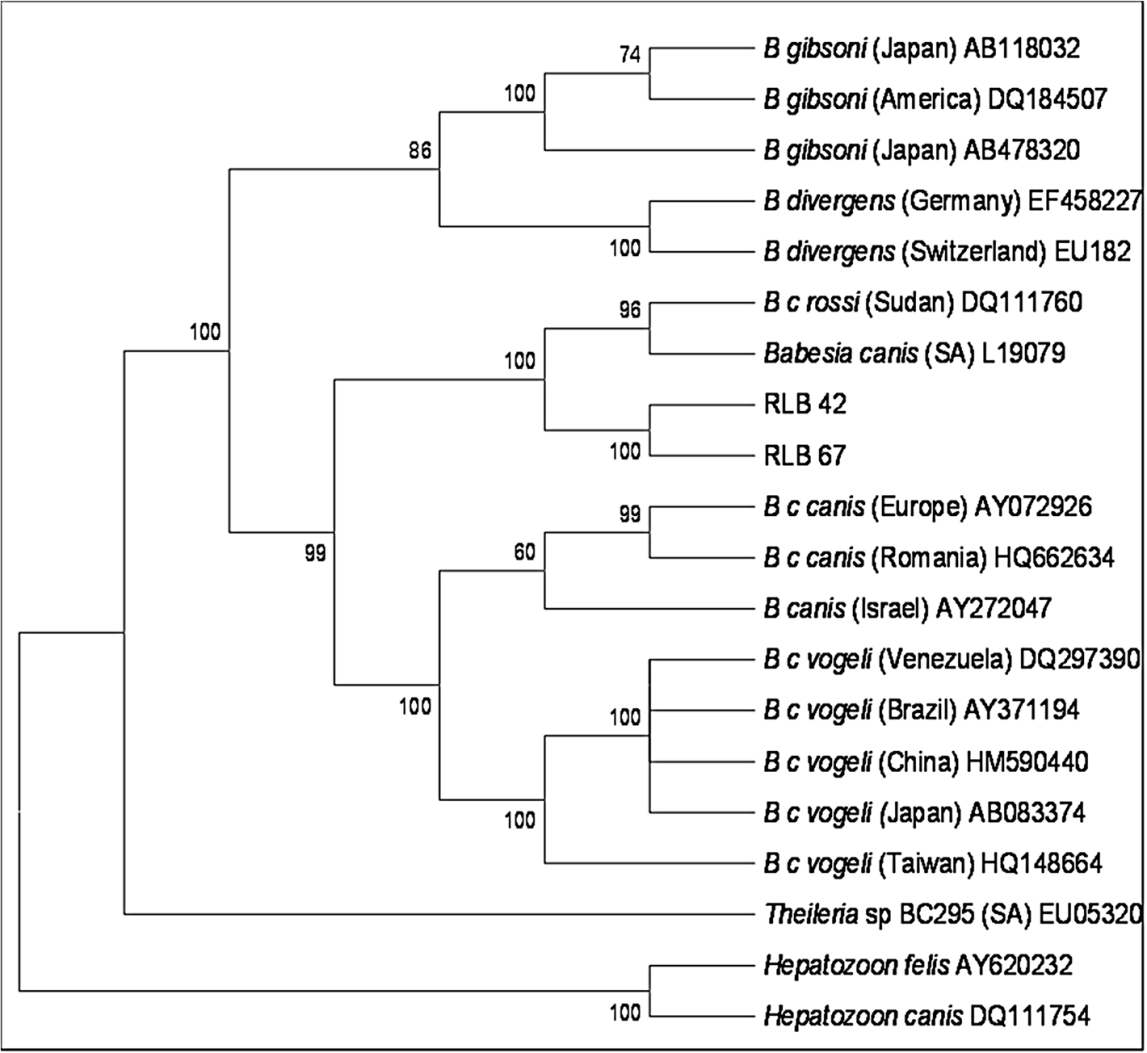
**Neighbor-joining tree, with the Kimura two-parameter distance **[47]**calculation showing the phylogenetic relationship of RLB 42 and 67 to related species based on the 18S rRNA gene sequences.** Relationships are presented as an unrooted tree with branch lengths being proportional to the estimated genetic distance between the strains.

Near-full-length 18S rRNA gene sequences were obtained from samples RLB42 and RLB67. A BLAST search revealed both samples showed a 99% similarity with *B. rossi*, a South African isolate (Accession number: L19079.1) and with *B. rossi* from Sudan (Accession number: DQ111760.1). The observed sequences were phylogenetically analysed to confirm their similarities. A neighbor-joining phylogenetic analysis was used to reveal the relationships between the generated 18S rRNA gene and other related *Babesia* and *Theileria* species. The analyses showed that the RLB42 and RLB67 sequences were closely related to *B. rossi* from South Africa and Sudan (Figure 1).

The pooled ticks collected from the dogs in this study are listed in Table 3. Larvae of *Cordylobia anthropophaga* were also encountered.

**Table 3:** List of ticks collected

**Species**	**Nymph**	**Adult**		**Total**
		**Male**	**Female**	**(%)**
*Amblyomma variegatum*	-	1	-	1 (1%)
*Haemaphysalis elliptica*	-	1	-	1 (1%)
*Haemaphysalis leachi*	-	4	23	27 (18%)
*Rhipicephalus lunulatus*	-	-	1	1 (1%)
*Rhipicephalus muhsamae*	-	1	2	3 (2%)
*Rhipicephalus sanguineus*	41	25	41	107 (73%)
*Rhipicephalus senegalensis*	-	1	2	3 (2%)
*Rhipicephalus turanicus*	-	3	-	3 (2%)

## Discussion

The main objective of this study was to use molecular techniques such as PCR/RLB to screen for the presence of tick-borne pathogens in a dog population in Nigeria. In addition, we aimed to determine whether the *BrEMA*1 gene, which has been postulated to be associated with virulence in *B. rossi-*infected dogs, was present [19]. The results of this study indicate the presence of a wide range of tick-borne pathogens circulating among the sampled dog population of Jos, Nigeria. The RLB results showed that 72% of the dogs screened harboured one or two tick-borne haemoparasites. The most common parasite recorded in this study was *B. rossi* (53%). The presence of *B. rossi* in dogs in Nigeria had been reported previously, but the prevalences found were much lower than the 53% that we report: 8/400 (2%) in a general survey [12] and 12/181 (6.6%) in dogs presented to veterinary hospitals [17]. At the Onderstepoort Veterinary Academic Hospital in South Africa, around 12% of all sick dogs presented at the outpatients clinic are diagnosed with babesiosis [32]. We found a single dog positive for *B. vogeli*, which confirms the very low prevalences reported in other studies: 1/400 (0.25%) [12] and 1/181 (0.6%) [17]. This is interesting since the known vector, *R. sanguineus*, was the most numerous tick encountered in our study. While *H. leachi* (sensu stricto) was the second most abundant tick encountered, we found a single *H. elliptica*, the proven vector of *B. rossi* in South Africa. This suggests that *H.* leachi may also be a competent vector of *B. rossi* in Nigeria*.*

To our knowledge, these are the first reports of the possible occurrence in dogs of *Theileria equi*, *Theileria* sp. (sable)*, Ehrlichia ruminantium* and *Anaplasma* sp. (Omatjenne) in Nigeria. The only *Theileria* sp. currently known to cause severe disease in domestic dog is *T. annae*, which was originally reported to occur in Spain [33,34]. *Theileria annae* has also been reported in North America, Portugal and Sweden [35-39]. *Theileria equi* and *T. annulata* have also been isolated from dogs in Spain [34,40]. The clinical significance of *T. equi* and/or *T. annulata* parasites in dogs remains unknown. *Theileria* sp. (sable)*,* possibly found in 9 dogs in our study, is known to cause mortalities in sable antelopes, but is also reported from other species where it appears to be an incidental finding [41]. A parasite similar to *Theileria* sp. (sable), designated *Theileria* sp. (dog), has been reported as an incidental finding in South African dogs [42], while a single dog from Jos South, Nigeria, was reported to be positive for *Theileria* sp. [17]. Parasites known to be virulent in particular hosts may infect accidental hosts without causing disease [40]. This may also apply to *E. ruminantium* and *A*. sp. (Omatjenne) possibly detected in our specimens. Since *B. rossi* was the focus of our study, the presence of the DNA of the other haemoparasites found was not confirmed by DNA sequencing. This should be followed up to rule out possible contamination or misidentification.

The *BrEMA*1 gene appears to be unique to *B. rossi* as it is absent in *B. vogeli* and *B. canis* isolates investigated [19]. At least 12 different *BrEMA1* genotypes have been reported in South Africa, of which 4 are associated with poor prognosis in *B. rossi*-infected dogs [19]. The negative results of the real-time PCR indicated that the *BrEMA1* gene was absent in all 38 specimens tested. This was surprising, but could possibly account for the absence of severe, complicated canine babesiosis cases in our sample. The question arises whether the *BrEMA1* gene is absent from *B. rossi* in other parts of Nigeria, as well as from other countries in sub-Saharan Africa.

In Nigeria, *Babesia* infections in dogs are usually mild and in some cases sub-clinical. This is contrary to the South African situation where *B. rossi* infections are often associated with clinical complications and mortalities. The reason for the virulence of the *B. rossi* isolates from South Africa may be due to the presence of wild canids, e.g., African wild dogs (*Lycaon pictus*) and black-backed jackals (*Canis mesomelas*), in the maintenance of the life cycle of *B. rossi*[43]. In Nigeria, wild canid populations have been persecuted to the point where there are no naturally occurring wild canids [44]. Given this fact, we can speculate that the life cycle of *B. rossi* is currently maintained only within the domestic dog population in Nigeria. This is contrary to the South African situation, where *B. rossi* has been detected and isolated from wild dogs. In addition *H. elliptica*, the known vector of *B. rossi*, has been collected from wild and domestic dogs in South Africa [45,46]. This may imply that *B. rossi* in Nigeria has through time and by natural selection lost its virulence, and by implication, the *BrEMA1* gene and its associated virulent genotypes. Thus, further studies will be required to investigate the role of wild canids in the maintenance of virulent *B. rossi* genotypes circulating within domestic dog populations.

Comparison of the *B.rossi* 18S rRNA gene sequences obtained indicates that the *B. rossi* sequences from Nigeria are closely related to the *B. rossi* from South Africa (Accession number: L 19079) and Sudan (Accession number: DQ 111760) with a 99% identity. Since the sequences are similar, this supports our conclusion that the *B. rossi* isolates circulating in domestic dogs in Nigeria are indeed true *B. rossi* (sensu stricto).

## Conclusions

At least 8 tick-borne pathogens possibly occurred in the sampled dog population at Jos, with *B. rossi* being the most prevalent. No severe or complicated cases of canine babesiosis were reported, suggesting either that the dogs cope fairly well with infection or that the *B. rossi* in that area is less virulent than elsewhere, e.g. in South Africa. The absence of the *BrEMA1* gene, which has been postulated as being linked with clinical outcome of infection, may possibly account for *B. rossi* occurring in that area being less virulent than South African isolates. The high prevalence of *B. rossi* infection indicates the presence of a competent vector of this pathogen; circumstantial evidence points to *H. leachi* (sensu stricto) being the vector.

### Ethical consideration

This research was approved by the Research Committee of the College of Veterinary Medicine, University of Agriculture, Makurdi, Nigeria.

## Competing interests

The authors declare that they have no competing interests.

## Authors’ contributions

MA participated in designing the study and wrote the first draft; DOO collected the specimens; MT and DPM performed the laboratory work and did the phylogenetic analysis; BLP wrote the final draft of the manuscript; PTM supervised the project. All authors read and approved the final version of the manuscript.
